# Supplementary material for: Genome-scale metabolic modeling of Ruminiclostridium cellulolyticum: a microbial cell factory for valorization of lignocellulosic biomass
Source: mSystems. 2025 Sep 30;10(10):e00960-25. doi: 10.1128/msystems.00960-25 (PMC12542651; doi:10.1128/msystems.00960-25)
Supplement: Supplemental material — Supplemental figures and tables. [file msystems.00960-25-s0001.pdf]

Supplementary material

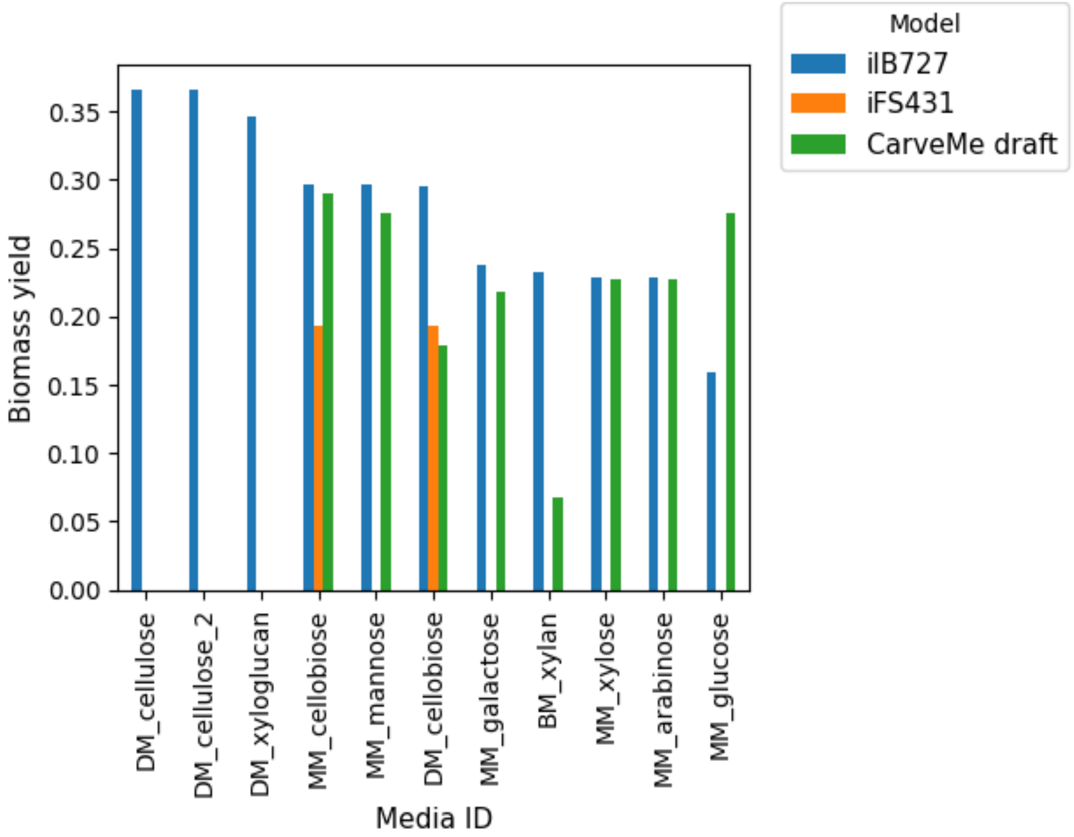

Figure S1: The biomass yield (gDW/g substrate) on multiple media as predicted by the three different models.

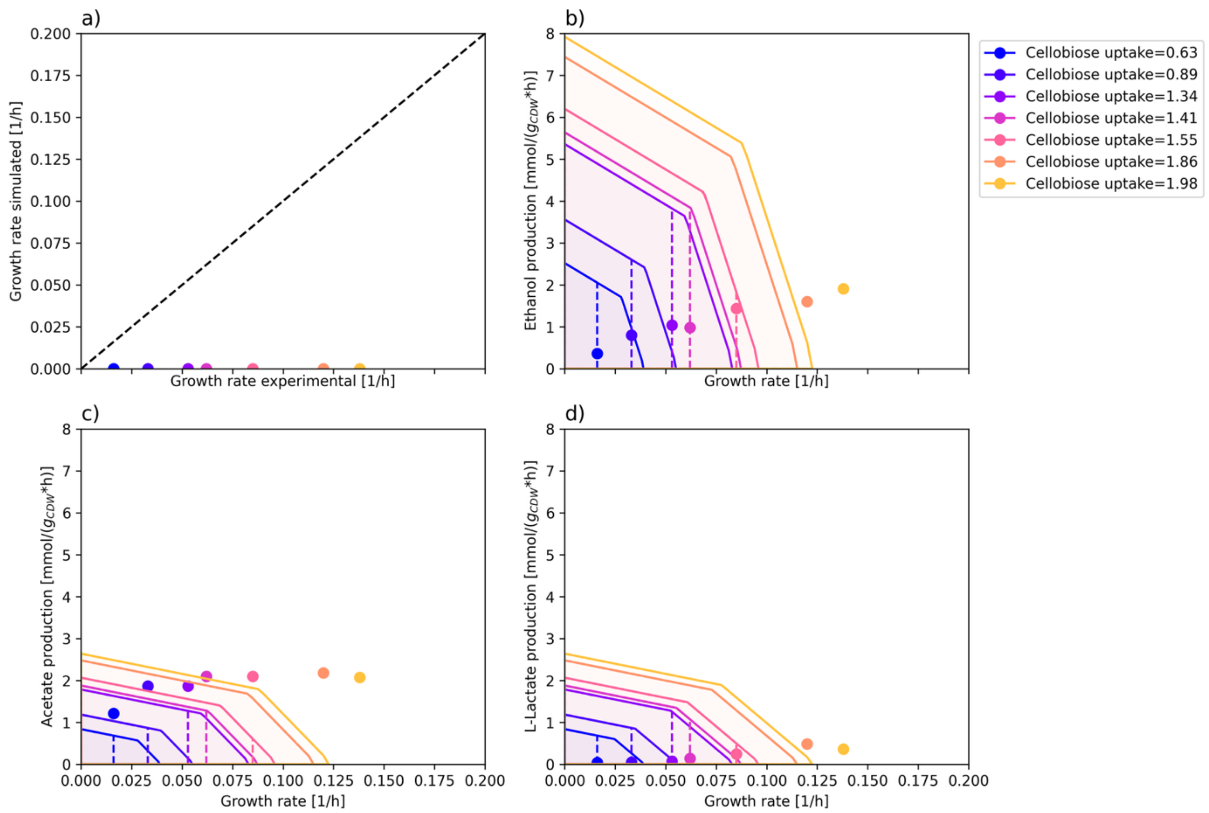

Figure S2 a) Experimental vs simulated growth rates with CarveMe draft model (with constraints on fermentation product production, causing inability to grow). **b-d)** Production envelopes of the main fermentation products: acetate, ethanol, and L-lactate. The ranges are obtained with flux variability analysis in a carbon-limited scenario using the experimentally measured cellobiose uptake rate at various dilution rates. The circles indicate the measured production rates of each product at different growth rates, whereas the dotted lines show the flux variability at each growth rate.

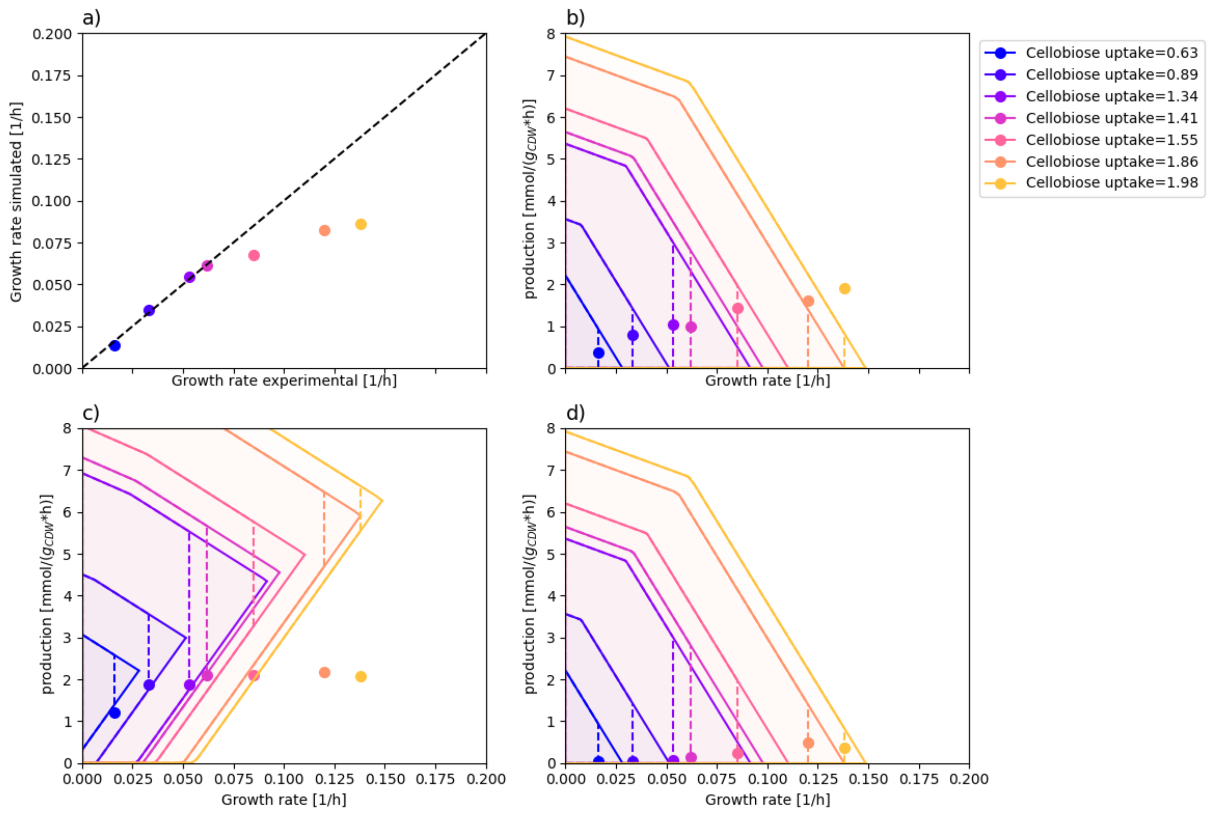

Figure S3 a) Experimental vs simulated growth rates with iFS431 model (with constraints on fermentation product production). The circles indicate the experimental and simulated growth rate at each uptake rate of cellobiose. **b-d)** Production envelopes of the main fermentation products: acetate, ethanol, and L-lactate. The ranges are obtained with flux variability analysis in a carbon-limited scenario using the experimentally measured cellobiose uptake rate at various dilution rates. The circles indicate the measured production rates of each product at different growth rates, whereas the dotted lines show the flux variability at each growth rate.

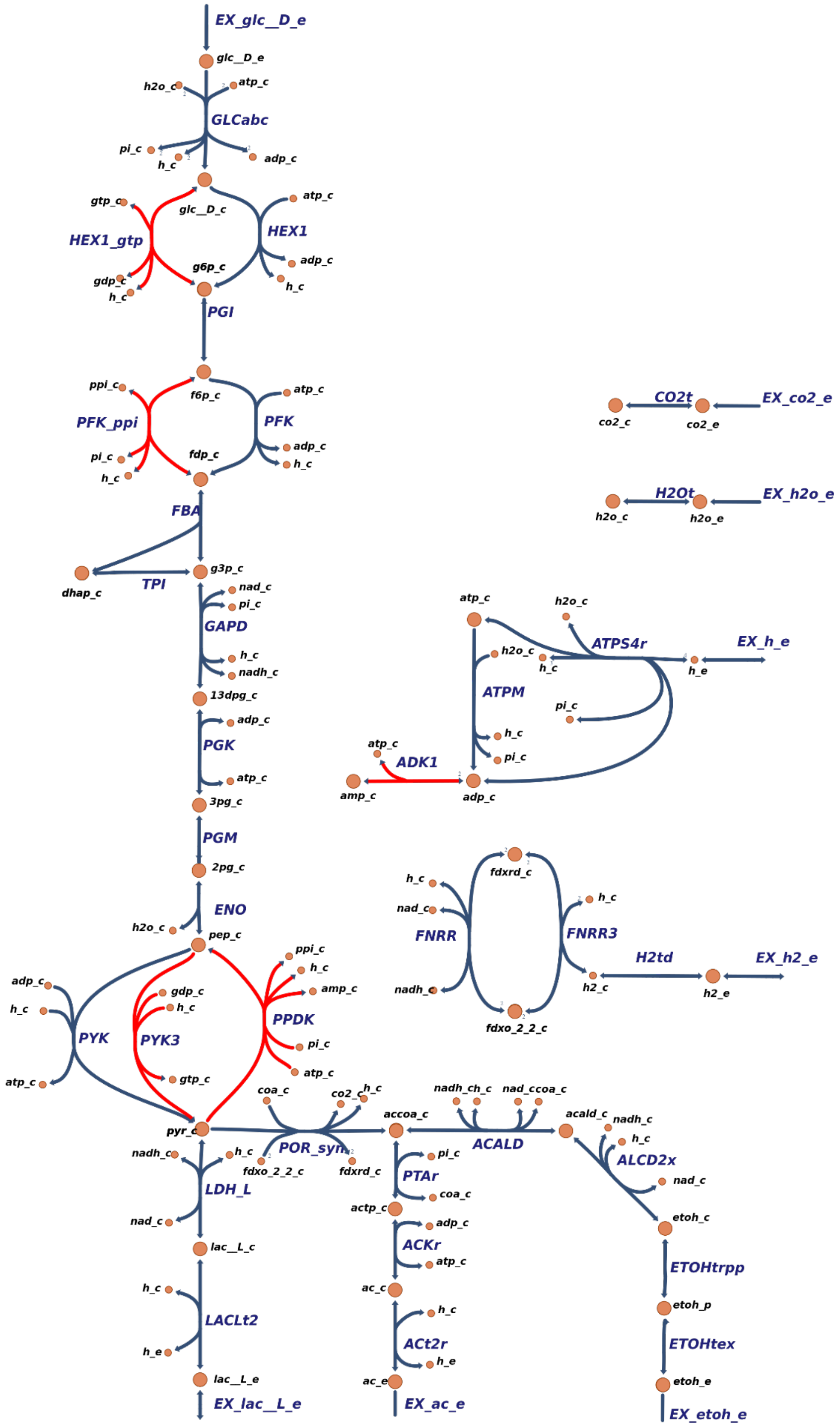

Figure S4: Pathway map including metabolic reactions of the central carbon model. Red reactions show the reactions that were not present in the ATP-based model but were present in the GTP/PPi-based model. The reactions that are not present in the GTP/PPi-based model are HEX1 and PFK, because they were replaced by reactions with the correct cofactor usage.

Table S1: Results of the maximization of fermentation products using the two variations of the central carbon model and the genome-scale model.

Objective (max)	Core model 1 (GTP and PPi)	Core model 2 (ATP)	iIB727
**Acetate production**	20.0	20.0	20.0
**Ethanol production**	20.0	20.0	20.0
**L-Lactate production**	20.0	20.0	20.0
**ATP production (ATPM)**	20.0	20.0	20.0

Table S2: Parameters for dFBA simulation for growth on cellulose with iIB727. Allowed range is represented by the allowed range for the parameter estimation with differential evolution.

Parameter	Value	Optimized	Allowed range
$v_{max,glc}$	8.42	Yes	(0.1, 10)
Km_{glc}	1.34	Yes	(0.1, 2)
$v_{max,cellb}$	9.59	Yes	(0.1, 10)
Km_{cellb}	1.26	Yes	(0.1, 2)
$v_{max,cellulose}$	4.06	Yes	(2.9, 5)
$Km_{cellulose}$	4.4	No	.
Ki	11	No	.

Table S3: Glucose equivalents for the oligosaccharides used in the simulation of degradation of lignocellulosic mixtures.

Polysaccharide	Oligosaccharide	Glucose equivalents
Arabinoxylan	AX	2.5
Arabinoxylan	AXX	3.333
Arabinoxylan	A23XX	4.167
Arabinoxylan	XAXX	4.167
Arabinoxylan	XA23XX	5.0
Xyloglucan	QLQG	7.5
Xyloglucan	QQLG	7.5
Xyloglucan	QLLG	8.5
Xyloglucan	QQQG	6.5
Xyloglucan	GQQG	5.667
Cellulose	Cellb	2.0
Cellulose	Cell3	3.0
Cellulose	Cell4	4.0
Cellulose	Cell5	5.0
Xylan	Xylb	1.667
Xylan	Xyl3	2.5
Xylan	Xylan4	4.333
Xylan	Xylan8	8.667
